# Knowledge, attitudes and skills in melanoma diagnosis among doctors: a cross sectional study from Sri Lanka

**DOI:** 10.1186/s13104-018-3499-y

**Published:** 2018-06-14

**Authors:** H. M. M. T. B. Herath, B. S. D. P. Keragala, W. A. E. Udeshika, S. S. M. Samarawickrama, S. P. Pahalagamage, Aruna Kulatunga, Chaturaka Rodrigo

**Affiliations:** 1 National Hospital of Sri Lanka, Colombo, 01000 Sri Lanka; 2 School of Medical Sciences, University of New South Wales, Sydney, 2052 Australia

**Keywords:** Malignant melanoma, Knowledge, Attitudes, Skills, Sri Lanka

## Abstract

**Objectives:**

This study aimed to assess the knowledge, attitudes and skills of non-specialist doctors on timely referral of suspicious lesions for melanoma diagnosis.

**Results:**

One hundred and twenty-three doctors (mean age; 30.4 years, SD ± 8.015) were enrolled. Very few (3.3%) correctly stated all four types of melanoma. Only 8.1% of the total sample had been trained to perform a total body examination for skin cancer detection and a majority (110/123) had never performed one. Almost all (95.2%) were not confident in using a dermatoscope for examination of a skin lesion. Only 17.9% of participants had discussed skin cancer/melanoma risk reduction with patients. Only 13.8% had educated at least one patient regarding skin self-examination for suspicious skin lesions. Knowledge and clinical skills regarding melanoma recognition was unsatisfactory in our sample. Urgent attention is needed to bridge the gap in knowledge and clinical skills on this topic.

## Introduction

The global incidence of malignant melanoma has increased but mortality has remained stable [1]. In Sri Lanka, the incidence was 0.33 cases per 100,000 in 2010 [2]. Most cases of malignant melanoma, if detected at an early stage, can be cured with surgical excision. Despite the new treatment modalities, survival of advanced melanoma with distant metastasis has not changed significantly over the last decade [3]. Therefore, it is essential to minimize delays in diagnosis to improve patient outcomes.

Previous studies have shown that physician related factors such as unawareness, misdiagnosis, use of inappropriate diagnostic methods, delayed referral and complacency about suspicious lesions may lead to diagnostic delays [4–6]. Misdiagnosis is common with atypical cases or melanomas occurring in less visible areas of the body. A retrospective analysis of 83 patients found that 52% of subungual melanomas and 20% of palmoplantar melanomas were clinically misdiagnosed by physicians. Non-dermatologists were responsible for 85% of these misdiagnoses [7]. Other studies had observed longer delays in diagnosis and treatment in patients with less apparent lesions, particularly on the head, neck and back [5, 8].

Diagnostic delays adversely affect patient survival [4, 5, 7–11]. In a study on acral melanomas, increased time to diagnosis was associated with increased tumour thickness, advanced stage of disease and a lower 5-year survival [7]. In superficial spreading melanoma and nodular melanoma, diagnostic delays had a significant positive correlation with Clark’s level of invasion and with tumor thickness [8].

The delay on part of medical professionals is partly due to lack of training. A study in seven medical schools and four residency programs in the United States showed that 75.8% of doctors were never trained and 55.3% had never observed a skin cancer screening examination [12]. In Australia, the sensitivity of melanoma detection by skin examination was lower for primary care clinicians in general practice compared to those practicing in skin cancer clinics [13]. Appropriate training has shown to improve the diagnostic accuracy and timely referral among doctors [14].

As mentioned previously, Sri Lanka has a relatively low incidence of all skin cancers including melanoma. In the undergraduate medical curriculum, not much emphasis is given for dermatological malignancies. Given these observations, this study aimed to assess the knowledge, attitudes and skills of non specialist doctors (excluding those working in surgical, oncology and dermatology units) on melanoma diagnosis and timely referral.

## Main text

### Methods

We conducted a descriptive, cross sectional study among doctors working in National hospital of Sri Lanka (NHSL), which is the premier healthcare institution of the country. It also the main tertiary care referral center in the country.

The study population comprised of non-specialist doctors working in the General Medical clinics and wards as well as in the outpatient department of NHSL. Non-specialists at different stages of their career were included if they were involved in patient care. These included; intern medical officers, resident medical officers, senior house officers and postgraduate trainees. Doctors attached to dermatology, oncology and surgical units were excluded as their work involves routine exposure to patients with skin malignancies or suspicious lesions. The data collection instrument was a self-administered questionnaire which included questions on knowledge, attitudes and practices (KAP) with regard to diagnosis of melanoma. The knowledge component was tested with questions regarding types of melanoma, risk factors, clinical features, course of the illness, current treatment methods, criteria for referral and prognosis, while the questions testing attitudes focused on self perceived importance of melanoma as a potential diagnosis in at-risk patients, and self perceived importance and willingness to carry out a total body examination, appropriate referrals and health education for at-risk patients. The practice component was tested by questions on familiarity with a dermoscope, number of total body examinations performed, number of patients that had received health education from each doctor and number of at-risk patients referred to specialist care. The latter three indicators were recorded for the preceding 12 months. Most questions were of true/false, yes/no format or required to input a number (or to select from multiple choices) as the answer. The answers to attitudes questions were based on a likert scale ranging from 1 (least important or least confident) to 5 (most important or very confident). The content knowledge was assessed based on the guidelines published in 2010 by the British Association of Dermatologists [15]. The questionnaire was pre-tested on 10 intern medical doctors.

The data analysis was carried out with SPSS statistical software (Version 23, IBM, USA). Findings relevant to descriptive statistics were summarized into proportions and averages based on the scales of measurements. Ethical approval for the study was obtained from the ethics review committee of NHSL.

### Results

One hundred and twenty-three doctors (mean age; 30.4 years, SD ± 8.015) working in NHSL, who met the inclusion criteria, were assessed (response rate: 61.5%). Only 4 out of 123 (3.3%) correctly stated all four types of melanoma and 99 (80.5%) could not name any. However, 102 (82.9%) doctors were aware of at least two risk factors for melanoma. Lymph nodes were correctly recognized as the mostly likely site for initial metastasis by 53 (43%) doctors. However, only 13.8% correctly stated that the lung is the most likely site for visceral metastasis. Tables 1 and 2 summarize the percentages of correct responses to questions that tested on the knowledge component.

**Table 1:** Responses of doctors (n-123) regarding melanoma awareness

Question	True^a^	False	Don’t know
The radial growth phase may not be evident in some melanomas	52.8	3.3	43.9
Melanoma is the most serious skin cancer	58.5	19.5	22
The incidence of melanoma is rising	71.3	22.1	6.6
Some melanomas can be amelanotic melanoma	69.9	0	30.1
Some melanomas can occur on the palms, soles and subungual areas	62.6	12.2	25.2
Melanoma can develop from the mucosal epithelium that lines the respiratory, gastrointestinal and genitourinary tracts	48.0	13.8	38.2
If detected early, melanoma can be cured with surgical excision	85.4	0.8	13.8
Dermoscope allows visualization of the skin structures in the epidermis, dermo-epidermal junction and the upper dermis	25.2	8.1	66.7
The diagnosis of melanoma is confirmed by excision biopsy	68.3	10.6	21.1
An elevated LDH at diagnosis or at follow up visit may indicate distant metastasis	34.1	7.3	58.6

^a^ The correct response to all questions is “True”

**Table 2:** Responses of doctors in the sample (n-123) regarding criteria for referral to a specialist

Question	True	False	Don’t know
A new mole appearing after the onset of puberty, which is changing in shape, colour or size	70.7	10.6	18.7
A long standing mole which is changing in shape, colour or size	80.5	4.1	15.4
Any mole which has three or more colours or has lost its symmetry	65.0	5.7	29.3
A mole which is itching or bleeding	60.2	9.8	30
Any new persistent skin lesion, specially if growing, pigmented or vascular in appearance and if the diagnosis is not clear	74.0	5.7	20.3
A new pigmented line in a nail specially where there us associated damage to the nail	42.3	21.1	36.6
A lesion growing under a nail	54.5	7.3	38.2

Regarding practices related to melanoma diagnosis, only 10 (8.1%) had received formal training to perform a total body examination for skin cancer detection. A majority (110, 89.4%) had never done one in their career. Out of the 13 doctors who have performed total body examination only 2 (15.3%) have done it more than five times in the preceding 12 months. The average score on the likert scale that assessed confidence regarding total body examination was 2.09 (SD: ± 1.05). The score on self-perceived confidence in diagnosing melanoma was 2.23 (SD: ± 1.1). Almost all (117, 95.2%) were not confident in using a dermoscope for examination of a skin lesion.

Only 22 (17.9%) and 17 (13.8%) doctors had discussed regarding skin cancer/melanoma risk reduction and self examination of skin with patients (respectively). A majority (90, 73.2%) stated that they were not confident in educating a patient regarding skin (self) examination. The mean value in the likert scaling system for this question was 1.84 (SD ± 1.05). Most of the doctors (105, 85.4%) in our sample had never referred a patent to a dermatologist in the preceding 12 months for suspected melanoma, 17 (13.8%) had referred less than five such patients and one doctor had referred between five to ten patients.

Regarding attitudes, most (86, 69.9%) agreed with the statement that doctors should be trained to perform a total body skin examination for skin cancer detection. A majority (93, 75.6%) were happy to attend a training program on performing a total body examination and identifying suspicious skin lesions.

### Discussion

This descriptive cross-sectional study on knowledge, attitudes and practices of non-specialist doctors regarding melanoma in a tertiary care hospital in Sri Lanka found that the majority of respondents did not have a satisfactory level of knowledge or training.

Promoting early diagnosis by educating general population as well as doctors is a widely accepted strategy to improve melanoma prognosis [16, 17]. The incidence of malignant melanoma in Sri Lanka [2] and in rest of South and Southeast Asia are low which probably explains the low priority given for the topic in undergraduate medical education. However, with the end of the civil conflict in Sri Lanka, more tourists and foreigners are visiting Sri Lanka who fall into of the category of high risk skin phenotype for melanoma. Some of them reside in the country for months if not years and it is important that local doctors are capable of catering to the needs of the visitors. A significant number of doctors also migrate overseas [18] or go for short-term clinical training to countries with a high incidence of skin cancer [19, 20]. Better awareness and clinical competence in suspecting and diagnosing melanomas is therefore essential for Sri Lankan doctors.

The lack of awareness and clinical skills regarding of skin cancer diagnosis has been reported in high incidence countries as well [12, 21, 22]. Reasons for the knowledge and practice gaps identified in these studies include lack of time, lack of training and confidence. The results were very similar in our study. In Sri Lanka, during the five and half year undergraduate medical training, the time allocated for dermatology appointment is between 2 and 4 weeks. After that, unless they choose a postgraduate career in dermatology, opportunities for further training are minimal or non-existent. Most respondents in this study acknowledged the need for further training on this topic.

Dermoscopy is a useful tool in evaluating skin lesions which can help to differentiate a benign pigmented lesion from a potentially malignant one. This is a noninvasive technique which is not popular in primary health care settings probably due to lack of training and awareness. A meta-analysis of studies that compared dermoscopy guided examination as opposed to naked eye examination concluded that dermoscopy is more accurate and sensitive in detecting melanoma [23]. Only one quarter of our sample was aware of dermoscopy and its advantages. A majority (118, 96%) were unskilled in using one. However, at the same time, it should be appreciated that even trainee dermatologists need time to master dermoscopy and in the hands of non-specialists, the rates of false positives can be high. Therefore, appropriate recommendations on this topic should be in the hands of specialist colleges and the panels responsible for post-graduate and continuous medical education programmes.

Primary care clinicians should have a relatively low threshold for referring a patient with a suspicious lesion to a dermatologist to determine if biopsy is indicated. Unfortunately, evidence from high incidence countries suggest that primary care physicians may fail to refer potentially malignant skin lesions in a timely fashion [24]. In this study, a majority of doctors had never referred a patient with a suspicious lesion to a dermatologist.

Currently the role of community based screening for skin cancers (including melanoma) is unclear [25]. One analysis in USA, suggested that one-time melanoma screening for the general population over 50 years of age and biannual (every 2 years) screening for first degree relatives of melanoma patients would be cost-effective [26]. Few studies have shown that skin self-examination lead to early diagnosis with the detection of thinner tumors [27–30]. In Sri Lanka, there are no guidelines or data to recommend periodic skin examination. In this study, majority of the doctors were not confident in performing such an examination by themselves or in educating patients on how to perform a self examination. Not surprisingly, many of them have never advised or educated patients regarding melanomas. Given these findings we believe educating doctors and medical students on common skin malignancies and full body (skin) examination will have a long standing impact on patient care. Several studies in other countries have shown significant improvements in knowledge, confidence and clinical practice of doctors following such training programmes [31–33].

### Conclusion

Overall, the knowledge and practices of doctors regarding melanoma was unsatisfactory in our sample. This is probably due to inadequate teaching and training opportunities during undergraduate and postgraduate medical education for students and trainees in non-dermatology or non-oncology fields. However, the attitudes were positive in acknowledging the knowledge gap and demonstrating willingness to engage in continuous medical education activities for improvement. Noting these facts, dermatologists and medical educators need to act to bridge this knowledge gap.

## Limitations

Regarding limitations of this study, we only included practicing doctors in the NHSL which has a disproportionately higher number of trainee doctors engaged in postgraduate courses and other continuous medical education activities. Though the level of awareness and response rate observed here is not representative of other peripheral hospitals in the country, it is unlikely that the findings will improve.

## Abbreviations

NHSL: National hospital of Sri Lanka
USA: United States of America
